# Help-seeking intention and associated factors towards mental illness among residents of Mertule Mariam town, East Gojam Zone, Amhara Region, Ethiopia: a mixed-method study

**DOI:** 10.1186/s12991-020-00261-y

**Published:** 2020-02-26

**Authors:** Berhanu Yeshanew, Asmare Belete, Mogesie Necho

**Affiliations:** 1 Department of Psychiatry, College of Medicine and Health Science, Dire Dawa University, Diredawa, Ethiopia; 2 Department of Psychiatry, College of Medicine and Health Science, Wollo University, Dessie, Ethiopia

**Keywords:** Help-seeking intention, Mental illness, Ethiopia

## Abstract

**Background:**

About 76% and 85% of people in low and middle-income countries with severe mental illness did not get management because of fear of expected discrimination. Studying the intention to seek help for mental illness will, therefore, help to know their intended plan for help that would have a vital role to access patients with mental illness. Despite this, literature is limited in the area and community-based studies are scarce in Africa in general and Ethiopia in particular concerning help-seeking intention towards mental illness and its associated factors. Therefore, we assessed the pattern of intention to seek help and associated factors for mental illness among residents of Mertule Mariam town that would fill the gap in evidence and serve as baseline information for public health intervention.

**Methods:**

A community-based cross-sectional study design was conducted from May to June 2017 at Mertule Mariam town. General Help-Seeking Questionnaire (GHQ) was used to assess the intention of help sought. Focus group discussion had also been employed to obtain qualitative data. A multi-stage sampling technique was used to obtain a total sample of 964 participants. Data were fed into Epi Info 7 and analyzed using SPSS version 21. The binary logistic regression method was used and an odds ratio with its 95% confidence interval was computed. Variables in multi-variable logistic regression were considered as an independent predictor of help-seeking intention to mental illness if their *P* value was less than 0.05.

**Result:**

About 81.5% of respondents had the intent to seek help from healthcare workers. But 44.6% of participants had the intention to seek from traditional healers. Variables that had an association with help-seeking intention were having an idea that mental illness needs treatment (AOR = 3.42, 95% CI 1.1–10.55), age group of 25–34 years (AOR = 1.46, 95% CI 1.02–2.09), mild social support (AOR = 1.85, 95% CI 1.25–2.72), and perceived severity of mental illness.

**Conclusion:**

Community help-seeking intent for mental health problems was still inadequate. So strengthening to deliver information about mental illness through media like radio and television to advance help-seeking intention of the community was mandatory.

## Background

Mental illness impinges on the individual’s ability to realize the capacity, handle normal life stressors, perform profitably and contribute to the society that he was living [1]. It is a great deal cause of disability-adjusted life years (DALY) [2]. Worldwide, mental and related diseases accounted for 13% of diseases’ load and expected to be 15% by 2020. And also in low and middle-income countries (LMIC), behavioral disorders attribute huge figures of disability. It contributes 25.3% and 33.5% among all the years lived with disability in LMIC, respectively [3].

In Ethiopia, mental illness is the foremost burden of illness among non-infectious disease including the rural parts of the country that accounts for up to 11% of illness burden exceeding the burden due to HIV/AID [4]. WHO reports showed that out of the clients who visited the psychiatric hospital at the outpatient department, 41% had a diagnosis of schizophrenia, 13% had a mood disorder and 11% were diagnosed with other disorders. Among patients who had got inpatient service, 85% have schizophrenia and mood disorder diagnosis [5].

Globally, the mental health treatment gap is huge. Up to 85% and 35–50% of clients in LMICs and high-income countries with severe mental health problems never receive treatment, respectively [6]. Henderson also supports that about 70% of the world clients with mental illness never receive treatment from mental health care staff [7].

Even though mental illnesses are highly prevalent and incapacitating to the society of this world [8], still no more help was demanded from the modern facility by society. Only 72.7% responded to seek help from healthcare workers as conditional with specification by ‘*if necessary*’. Among the reasons attributed by those who did not respond seeking help, 20.1% do not desire others to know their problem while 19.4% of them claim as they did not know where to seek help [9].

The society prefers habitual healers, as well as herbal treatments including holy water and community, took the patients with a mental health problem to modern medical centers when they lost hope and progress after trying exhaustively from informal traditional methods [8, 10].

Also, in some areas, people left clients who have mental illness alone if he/she did not respond to informal help source and modern treatments. Based on the patient’s condition, he/she chose to live with the family getting his/her basic needs or wander around the street naked [11].

A community-based study done in India showed that fewer respondents (18%) claim as they might visit a psychiatrist if they have an emotional problem while a relatively higher number (35%) prefers habitual healer as a means of treatment for their mental health problem [12]. Another study conducted in Nigeria showed that patterns of help-seeking revealed that 46% of respondents prefer orthodox medical care as a primary source of help and 34% of respondents prefer spiritual healing and also 18% prefer informal help as the first-line source of help than modern medicine [13].

In our country Ethiopia too, there is a huge treatment gap for the treatment of the mental health problem. A study has been done in Butajira and showed that only 41% of society prefers seeking help from health institutions [14]. According to the study conducted at holy water site of Gebremenfes Kidus [10], out of 25 patients who participated in the study 21 patients, which accounts for 84%, had help-seeking behavior from traditional form of help like religious leaders, holy water, and from individuals who believed to have special power of knowing mental illness and prescribing traditional treatment for mental illness. Those individuals were known by the community to have ability to treat mental illness. A qualitative study done in Borana also supports the finding of the Axum study. The Borena societies have intentions seeking help according to the following. They may seek help initially from Borana wise men consultation followed by indigenous healers, then modern health facility which is the last option [11].

Contributing factors for the help-seeking intention of the mental health problem were attributed to being female, good perceived social health, higher mental health awareness, higher educational level [15], ethnicity, younger age and good attitude towards mental illness [10].

These mental illnesses affect the social, academic, occupational and recreational parts of functionalities victims, family members, and a whole community. Perceived causes, perceived help sought and the prognosis is crucial in implementing integrative health care to mental illness. So this study has a vital role in assessing help-seeking intention and associated factors of the community towards mental illness in Mertule Mariam town.

## Methods

### Study design and setting

This study assessed the magnitude of help-seeking intention and its associated factor for mental illness among residents of Mertule Mariam town. The study utilized both quantitative and qualitative methods and was conducted in Mertule Mariam town from May to June 2017. Mertule Mariam town is located 364 km far from Addis Ababa, the capital city of Ethiopia. The town has a total population of 12,082**(30)**, 6028 males and 6054 females. Regarding health facilities the town, has one hospital, one health center, and four private clinics. Moreover, the town has been geographically demarcated into two administrative kebele with 2848 households in kebele one and 2833 households in kebele two. The Mertule Mariam primary hospital provides multiple services like chronic care service, inpatient and outpatient services, ophthalmic services, maternal and child health series, Human Immune Deficiency Virus treatment, and prevention service. Mental health service is also one of the services delivered in this hospital. The psychiatric unit of the hospital has both outpatient service and inpatient service which are staffed by four BSc psychiatric professionals. According to the staff report, the traditional healers link the psychiatric patients to a psychiatric unit of Mertule Mariam Primary hospital.

### Study participants

The sample size had been estimated with a single population proportion formula. The assumptions taken into consideration during the estimation of the sample size include the magnitude of help-seeking intention 59% [14] from a previous Ethiopian study and *z*-value of 1.96, margin of the error to be tolerated to be 0.05, design effect of 2 and non-response rate of 10%. So the total estimated sample size was 794. However, we also considered sample size calculation for the associated factors of help-seeking intention towards mental illness. So in this case, we calculated sample size using stat calc of Epi Info version 7 by taking confidence interval = 95%, power = 80%, design effect = 2 and an odds ratio of 0.55 for family history of mental illness which yielded the highest sample, 964 which was the final sample size for this study. A total of 964 adult people in the town aged 18 years and above who were available at home during the data collection period were joined in this study.

A multi-stage sampling technique was employed. A systematic sampling method was also used to select households. If two or more adults were living in the households, to select the adult who participates in data collection, the lottery method was employed. The study excludes those who were severely ill due to any form of medical illness that prevents them from giving an interview.

### Measurements and data collection instruments

The dependent variable was help-seeking intention, whereas the independent variables include socio-demographic factors (age, sex, ethnicity, religion, marital status, educational status, occupational status, and family income), and illness perception factors. Data were collected by interviews using a semi-structured questionnaire by using the translated Amharic version of the questionnaire. General Help-Seeking Questionnaire (GHSQ) had been implemented for the assessment of help-seeking intention for their perceived mental illness [16]. In addition to this, Community Attitude Towards Mental Illness Inventory (CAMI) was utilized to assess community attitude towards mental illness [17]. The overall reliability of CAMI was *a* = 0.84. Moreover, the Mental Health Knowledge Schedule (MAKS), Oslo 3-item social support scale and illness perception questionnaire were employed to assess knowledge about mental illness, social support level of participants and illness perception about mental illness, respectively. The questionnaire was pre-tested on 5% (49) of the total sample size participants and we did not include the results of the pretest in the final analysis. Focus group discussion which had a member of religious leaders, health workers and community participants who were selected purposely was also conducted to obtain the qualitative part of the data. The principal investigator moderated the discussion of focus group discussion. Audio records and hand notes were used during the discussion. Data collectors and supervisors were trained for 2 days. The collected data were reviewed and checked for completeness daily.

### Data processing and analysis

The quantitative data were entered using Epi info 7th version and exported to SPSS version 21 for analysis. Data were explored using descriptive statistical measures. Bivariate and multi-variable binary logistic regression analyses were used. A *P*-value of less than 0.2 on bivariate logistic regression was used to screen variables to be entered into multi-variate logistic regressions.

Then independent variables with a p-value less than 0.05 on the final model were considered as determinants of help-seeking intention. The strength of the association has been illustrated by the odds ratio (OR) with its 95% confidence interval. Thematic analysis was also enrolled in the analysis of the qualitative part.

### Operational definition

Attitude: measured based on four subscales of CAMI; authoritarianism, benevolence, social restrictiveness, and community mental health ideology. Attitude scores are dichotomized by their mean score [17].

Good knowledge: was defined if the participants answer the knowledge questions greater than the mean score [18].

Good help-seeking intention: was defined if the participants intend to seek help from health workers for personal or family mental illness when they thought they have a problem.

Social support: was categorized as poor if the score is 3–8, moderate if between 9 and 11 and strong if an overall score was between 12 and 14 [19].

### Ethical consideration

Ethical clearance was obtained from the institutional review board of the University of Gondar College of medicine and health science and review committee of Amanuel Mental Specialized Hospital. Permission letter from the Mertule Mariam town administration was also requested and obtained so that distributed to the two kebele administrations before the starting of the study. Written consent was obtained from each participant after full information regarding the study was supplied. The name of the participants was not included in the questionnaire and therefore the information gathered from the participants was kept confidential.

## Result

### Socio-demographic characteristics of participants

Out of 964 study participants invited, 947 were interviewed and gave complete responses which give 98.23% response rate. Among these participants, 554 (58.5%) were males while 393 (41.5%) were females. The mean age ± SD of the respondents was 30 ± 8.29 years and ranges from 18 to 80 years. Regarding the marital status of the participants, 426 (45%) were single followed by married, 417 (44%). Most of the participants were Amhara by ethnicity, 944 (99.7%) and orthodox by religion, 935 (98.7%). Concerning the educational level of participants, 267 (28.2%) had a college diploma and above followed by 260 (27.5%) who attended secondary school education. Concerning social support of the participants, moderate, poor and strong social support accounted for 409 (43.2%), 375 (39.6%) and 163 (17.2%), respectively (Table 1).

**Table 1:** Socio-demographic distributions of participants among residents of Mertule Mariam town East Gojam Zone, Amhara Region, Ethiopia, 2017

Independent variable	Categories	Frequency	Percent
Age	18–24	241	25.4
	25–34	463	48.9
	35–44	193	20.4
	> 44	50	5.3
Sex	Male	554	58.5
	Female	393	41.5
Marital status	Single	426	45.0
	Married	417	44.0
	Divorced	85	9.0
	Widowed	19	2.0
Ethnicity	Amhara	944	99.7
	Other	3	0.3
Religion	Orthodox	935	98.7
	Other	12	1.3
Educational status	Unable to read and write	116	12.2
Educational level	Elementary school	126	13.3
	Secondary school	260	27.5
	College diploma	267	28.2
	Degree and above	178	18.8
Occupational status	Government employee	387	40.9
	Housewife	70	7.4
	Farmer	59	6.2
	NGO employee	45	4.8
	Merchant	168	17.7
	Student	152	16.1
	Other	66	7.0
Income	< 750	290	30.6
	751–1200	113	11.9
	> 1201	544	57.4

### Information and previous experience about mental illness

**Fig. 1:**
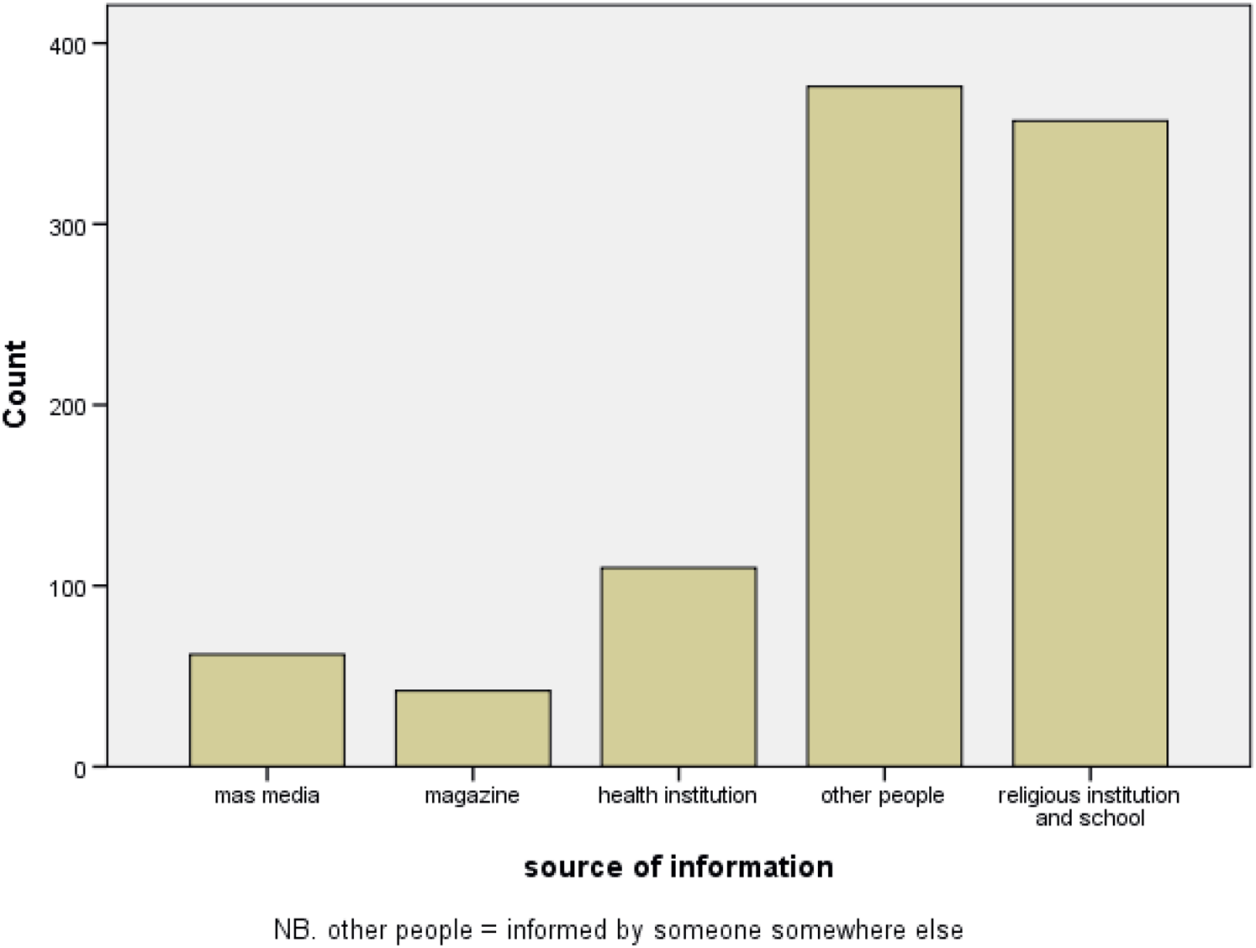
Sources of information about mental illness for residents of Mertule Mariam town, East Gojam Zone, Amhara Region, Ethiopia, 2017

About 575 (60.6%) of respondents have got information about mental illness during the last 1 year. Three hundred seventy-six (39.7%) of respondents got the information from other people while 110 (11.6%) got the mental health information from health care institutions. Moreover, about 62 (6.5%) of participants obtained information regarding mental illness from mass media, while 41 (4.4%) of participants got the information from magazines (Fig. 1). Regarding the previous experience of participants about mental illness, about 658 (69.5%) of them knew someone who had a mental illness. Six hundred sixty-three (70%) of respondents had never been involved in caring for someone who had a mental illness (Table 2).

**Table 2:** Previous experience about mental illness of participants of residents of Mertule Mariam town, East Gojam Zone, Amhara Region, Ethiopia, 2017

Independent variables	Categories	Frequency	Percentage
Have you ever suffered from mental illness?	Yes	91	9.6
	No	856	90.4
Do you know someone who has mental illness?	Yes	657	69.4
	No	290	30.6
If yes for the above question what is your relation?	Relative	65	6.9
	Neighbor	133	14.0
	Friend	144	15.2
	Other	605	63.9
Have you been involved in caring people who have mental illness?	Yes	284	30.0
	No	663	70.0
Have you ever been hurt by people who have mental illness?	Yes	315	33.3
	No	632	66.7
Did you witness someone hurt by people who have mental illness?	Yes	687	72.5
	No	260	27.5

Other: includes on street, other people’s home

### Knowledge, attitude and illness perception to mental illness

Despite the fact that 445 (47%) of participants had poor knowledge regarding mental illness, a significant portion of 307 (32.4%) and 550 (58.1%) of them level mental illness as severe and very severe, respectively. Majority 677 (71.5%) of clients believe different psychosocial factors as major causes of mental illness. Concerning the attitude of the respondents, 561 (59.2%) of them had community mental health ideology (Table 3).

**Table 3:** Attitude, knowledge and perception to mental illness among residents of Mertule Mariam town, East Gojam Zone, Amhara Region, Ethiopia, 2017

Variable	Categories		Frequency	Percent
Knowledge of mental illness	Good		502	53.0
	Poor		445	47.0
Perceived severity of MI	Mild		24	2.5
	Moderate		66	7.0
	Severe		307	32.4
	Very severe		550	58.1
Cause of MI	Psychosocial factor		677	71.5
	Nerve damage		13	1.4
	Poverty		22	2.3
	Substance use		102	10.8
	Evil spirit possession		59	6.2
	God’s punishment		71	7.5
MI needs treatment	Yes		773	81.6
	No		174	18.4
Place of treatment	Hospital		614	64.7
	Holy water		207	21.9
	Holy water and hospital		101	10.7
	Sorcerer		24	2.5
Attitude towards MI	Authoritarianism	Less	447	47.2
		More	500	52.8
	Benevolence	Less	495	52.3
		More	452	47.7
	Social restrictiveness	Less	580	61.2
		More	367	38.8
	Community MI Ideology	Less	386	40.8
		More	561	59.2

*MI* mental illness

### Help-seeking intention to mental illness

Among the total respondents, 772 (81.5%) were likely to seek help from healthcare workers while 423 (44.6%) of participated subjects were likely to seek help from traditional methods of treatment. Four hundred ninety (85%) respondents whoever got information about mental illness had the intention of seeking help from health workers. Regarding social support and the intent to seek help, 349 (85.3%) of those who had mild social support had the intention to seek help from health care workers. Fifty-six (90%) of those who got information from mass media and 98(89%) of those who got information from health workers had the intention to seek help (Table 4). Participants who perceived the severity of mental illness as mild 45 (68.2%) were less likely to have the intention of seeking help from a traditional source of help. Respondents who agreed that mental illness needs treatment; 341 (44.1%) had the intent to seek help from traditional medical practice (Table 5).

**Table 4:** Distribution of help-seeking intention for mental illness from health workers among residents of Mertule Mariam town East Gojam Zone, Amhara Region, Ethiopia, 2017

Variables	Category	Help-seeking intention from HW			
		Likely	Unlikely	COR	AOR
Age	18–24	194 (80.5%)	47 (19.5%)	Reference	Reference
	25–34	379 (81.9%)	84 (18.1%)	1.09 (0.73– 1.62)	1.31 (0.8–2.06)
	35–44	158 (81.9%)	35 (18.1%)	1.09 (0.67–1.77)	1.67 (0.9–3.0)
	> 44	41 (82%)	9 (18%)	1.10 (0.50–2.43)	2.27 (0.88–5.85)
Marital status	Single	360 (84.5%)	66 (15.5%)	Reference	Reference
	Married	325 (77.9%)	92 (22.1%)	0.64 (0.45–0.92)	0.48 (0.32–0.74)**
	Divorced	73 (85.9%)	12 (14.1%)	1.11 (0.57–2.16)	0.87 (0.41–1.82)
	Widowed	14 (73.7%)	5 (26.3%)	0.51 (0.18–1.47)	0.37 (0.11–1.28)
Social support	Poor	290 (77.3%)	85 (22.7%)	Reference	Reference
	Moderate	349 (85.3%)	60 (14.7%)	1.70 (1.18–2.45)	1.85 (1.25–2.72)**
	Strong	133 (81.6%)	30 (18.4%)	1.29 (0.81–2.37)	1.45 (0.88–2.37)
Source of information about mental illness	Mass media	56 (90.3%)	6 (9.7%)	Reference	Reference
	Magazine	34 (81.0%)	8 (19.0%)	0.45 (0.14–1.42)	0.41 (0.12–1.38)
	Health institution	98 (89.1%)	12 (10.9%)	0.87 (0.31–2.46)	0.79 (0.27–2.33)
	Other people	313 (83.2%)	63 (16.8%)	0.53 (0.22–1.28)	0.48 (0.19–1.22)
	Religious and school	271 (75.9%)	86 (24.1%)	0.33 (0.14–0.81)	1.02 (0.24–4.23)
Perceived severity of mental illness	Mild	15 (62.5%)	9 (37.5%)	Reference	Reference
	Moderate	56 (84.8%)	10 (15.2%)	3.36 (1.15–9.75)	0.35 (0.13–1.09)
	Severe	251 (81.8%)	56 (18.2%)	2.68 (1.12–6.45)	1.09 (0.51–2.30)
	Very severe	450 (81.8%)	100 (18.2%)	2.7 (1.15–6.34)	0.97 (0.66–1.42)
Do you think mental illness requires treatment?	Yes	629 (81.4%)	144 (18.6%)	1.88 (1.35–2.62)	3.42 (1.11–10.55)*
	No	143 (82.2%)	31 (17.8%)	Reference	Reference

*HW* health worker

*p-value < 0.05, **p-value < 0.01 *other people = informed by someone somewhere else

**Table 5:** Distribution of help-seeking intention towards mental illness from traditional help source among resident of Mertule Mariam town East Gojam Zone, Amhara Region, Ethiopia, 2017

Variable	Category	Help-seeking intention from TFOH			
		Likely	Unlikely	COR	AOR
Age	18–24	93 (38.6%)	148 (61.4%)	Reference	Reference
	25–34	226 (48.8%)	237 (51.2%)	1.51 (1.10–2.08)	1.46 (1.02–2.09)*
	35–44	81 (42%)	112 (58%)	1.15 (0.78–1.69)	1.03 (0.64–1.64)
	> 44	22 (44%)	28 (46%)	1.25 (0.67–2.31)	1.28 (0.61–2.71)
Marital status	Single	185 (43.4%)	241 (56.6%)	Reference	Reference
	Married	185 (44.4%)	232 (55.6%)	1.04 (0.79–1.36)	1.06 (0.76–1.48)
	Divorced	44 (51.8%)	41 (48.2%)	1.39 (0.87–2.23)	1.53 (0.89–2.62)
	Widowed	8 (42.1%)	11 (57.9%)	0.94 (0.37–2.40)	1.21 (0.41–3.54)
Social support	Poor	147 (39.2%)	228 (60.8%)	Reference	Reference
	Moderate	192 (46.9%)	217 (53.1%)	1.37 (1.03–1.82)	1.41 (1.04–1.91)*
	Strong	83 (50.9%)	80 (49.1%)	1.60 (1.11–2.33)	1.85 (1.24–2.75)**
From where you got the information?	Mass media	42 (67.7%)	20 (32.3%)	Reference	Reference
	Magazine	21 (50.0%)	21 (50.0%)	0.47 (0.21–1.06)	0.54 (0.23–1.26)
	Health institution	70 (63.6%)	40 (36.4%)	0.83 (0.43–1.61)	0.91 (0.45–1.83)
	Other people	169 (44.9%)	207 (55.1%)	0.38 (0.22–0.68)	0.37 (0.20–0.67)**
	Religious and school	120 (33.6%)	237 (66.4%)	0.24 (0.13–0.43)	0.44 (0.12–1.53)
How severe do you think mental illness is?	Mild	9 (37.5%)	15 (62.5%)	Reference	Reference
	Moderate	21 (31.8%)	45 (68.2%)	0.77 (0.29–2.06)	0.65 (0.26–1.61)
	Severe	130 (42.3%)	177 (57.7%)	1.22 (0.52–2.88)	0.43 (0.24–0.77)**
	Very severe	262 (47.6%)	288 (52.4%)	1.51 (0.65–3.52)	0.73 (0.54–1.00)
Do you think mental illness require treatment?	Yes	341 (44.1%)	432 (55.9%)	2.12 (1.62–2.78)	2.21 (0.73–6.66)
	No	81 (46.6%)	93 (53.4%)	Reference	Reference

*TFOH* traditional form of help

*p-value < 0.05, **p-value < 0.01 *other people = informed by someone somewhere else

### Factors associated with help-seeking intention to mental illness

On multivariable logistic regression age, marital status, social support, source of information, perceived severity of mental illness and perception about the need for treatment for mental illness were the associated factors for help-seeking intention. The odds of help-seeking intention among the respondents of Mertule Mariam town residents whose age was 25–34 years was 1.46 times higher for seeking help from traditional methods of treatment as compared to those participants whose age was 18–24 years (AOR = 1.46, 95% CI 1.02–2.09). Besides, participants who have moderate social support were 1.85 at higher odds of intention to seek help from health care workers than those participants who had poor social support (AOR = 1.85, 95% CI 1.25–2.72). But as the level of social support become strong, the increase in the chance of the individuals’ to have the intention to seek help from the traditional form of help; moderate social support (AOR = 1.41, 95% CI 1.04–1.91) and strong social support (AOR = 1.85, 95% CI 1.24–2.75). Moreover, participants who get information about mental illness from other people were 63% less likely to seek help from a traditional form of treatment (AOR = 0.37, 95% CI 0.20–0.67). Besides, respondents who thought that mental illness as severe were also 57% times less likely to seek help from traditional methods of treatment (AOR = 0.43, 95% CI 0.24–0.77) as compared to those participants who considered mental illness as mild. The odds of intention to seek help from health workers among participants who believed that mental illness needs treatment was 3.42 times higher than those participants who believed that mental illness does not need treatment (AOR = 3.42, 95% CI 1.11–10.55) (Tables 4 and 5).

### Qualitative descriptions

Four focus group discussions were held with a minimum group size of five and a maximum group size of six participants and were limited when the idea saturates starting from the third session (group). Most of the participants explain their idea as people who have mental illness are unable to do anything good for their lives and no more worthy they are for their family unless they bring insult to them.

### Stigma indicators

Most participants in the discussion agreed that mentally ill patients are aggressive and violent physically; they go naked and collect garbage. The fear of danger imposed by the mentally ill patient is cause of the stigma.

One group discussants raised their idea as follows:

*‘…..they lose their control, they go naked, and mostly they use substances like ‘tela’, ‘araki’ and bite people they got without any reason. They do not know anything because they are mad… they are mad (ebid)’.*

Another 32-year-old female discussant suggested that mentally ill people are dangerous physically if they are allowed to live in the same home.

*‘….mentally ill peoples are difficult to know their behavior and should be left outside unless they may kill their family members. If possible it may be helpful to take them to holy water and if they don’t have any improvement better to leave on the street…’.*

Their attitude is more of an avoidant and authoritarian type judging that they are substance users, prolonged sitters, and fighters for no reason. This idea may push family members of the mentally ill to either restraining patients in the home or leaving outside of the home on the street. On the treatment choice, they give the probability of helping only religious aspects and behavioral parts. These people may not take their family member who has a mental illness to the hospital for help.

### Perceived causes of mental illness

Most discussants present their perceived cause of mental illness as social and evil spirit possession related. Among the listed causes; possessed by evil spirit due to violation of God’s doctrines and testaments, attack by devil spirit, loss of loved one, poverty, too much thinking, and substance use are the commonest.

Discussants of different group discuss their idea regarding the cause of mental illness as follows:

*‘…mostly mental illness comes when the mother soon after she gave birth to a baby is left alone, substances are taken in large amounts, peoples could not get what they love. Peoples also get mentally ill when they try to steal ‘tabot’ and other materials of the church as punishment by God’s hand. Poverty exposes people to too much work time and too much thinking which is the main cause of mental illnesses’.*

### Possible treatment options

Almost all discussants prefer traditional methods of treatment as the first-line source of help like holy water, praying and herbal treatments including wise men.

One 38-year-old woman who has two children with a mental illness described her preference for help exclusively holy water and praying.

‘*…I have two male children who have mental illness and the elder have been treated in Amanuel Mental Specialized Hospital. He had had some improvement while he takes the medication but worsened when he stopped taking medication. I took him to holy water last year and he is now fine but his younger brother recently become mentally ill. I will take him to holy water soon. The hospital medication never helps anything for mental illness…’.*

Another member hesitates about the effectiveness of the treatment by modern medicine.

*‘… hospitals may help to calm patients just to relief from the destructive behavior of the patient but for permanent it seems good to take him to holy water …’.*

Another 29-year-old receptionist (hotel) supported the traditional treatment preference raising the issue of her elder brother who has been mentally ill 5 years ago.

*‘… I think the best and ultimate treatment of mental illness is taking to sorcerer unless the rest of the treatment methods are nothing. We took my brother to the hospital, holy water but nothing we get….’*

One male participant whose father had had mental illness but now healthy strongly argued that ‘*holy water and praying is the only treatment for mental illness…’.*

Another 25-year-old female radiographer argued the traditional methods of treatment as follows:

‘*…taking mentally ill people to magicians and sorcerers is duplicating the devil and it is good only to take them to the hospital…’.*

## Discussion

As per the knowledge of researchers of the current study, there is a limitation of evidence regarding the help-seeking intention of the community towards mental illness in developed countries in general and developing countries like Ethiopia in particular. This shortage of evidence makes the treatment of mental illness difficult and creates a huge gap for the unmet needs of the treatment for mental illness. Therefore, this study assessed help-seeking intention for mental illness and its associated factors that would be an important milestone in narrowing the gap for treatment of mental illness since it would serve as an evidence mental health intervention activities.

In this study, the pattern of good help-seeking intention for mental illness was obtained to be high (81.5%). A study done in Malaysia obtained that 72.7% of participants had intended to seek help from health care workers [9] which is relatively lower than the present study. Another study in India assessed help-seeking intention of community and found that 18% of the participants had an intention to seek from a psychiatrist [12] which was also lower than the current study. Moreover, the help-seeking intention from health care workers was 46% in Nigeria [20], 41% in Ethiopia [14], 67.7% in Japan [21] all of which are lower as compared to the present study. The justification for this might be because of the variations in socio-demographic factors as well as cultural and economic variations between countries where the current and previous studies were conducted. Moreover, methodological variations like the use of different assessment methods for help-seeking in current and previous studies could contribute to the discrepancy. This study has also evaluated the traditional help-seeking intention of the community. Around 44.6% of the participants had the intention to seek help from a traditional form of help for perceived mental illness. This finding had been less than a result reported by the study done in Japan; 75.6% and also the qualitative result of this study also favor help-seeking intention more from informal treatments.

Among the variables which have a significant association with intention to seek help from health workers for perceived mental illness in Mertule Mariam community were being married, having moderate social support, and having an idea that mental illness requires treatment. In this study, participants who were married were 52% less likely to seek help from health workers than participants who were single in marital status. Up to the knowledge of the investigators, this finding might be considered as new finding. The probable reason that hinders the married individual from seeking help from health workers might be the lack of knowledge about mental illness, and the single participants might be prone to information about mental illness.

Besides, having a moderate level of social support was 1.85 times at an increased level to have the intention to seek help for perceived mental illness from health care workers than participants who have poor social support. This finding was in line with another study done in Ethiopia, in which the presence of strong social support increases the intention to seek help from health professionals [22]. This means that the participants who have good social support had an increased chance of seeking help for their mental health problems from health workers. The other variable that had a significant association with help-seeking intention from health workers in this study was having the idea that mental illness requires treatment. This means that participants who have an idea that mental illness requires treatment were 3.42 times more likely to have the intention to seek help from health workers than those participants who have no idea that mental illness requires treatment. The reason might be that participants’ who think of treatment are required for mental illness had good knowledge that biological treatment is needed for the treatment of mental illness and therefore might have high intention for seeking help from health care professionals.

Moreover, in this study, age group of 25–34 years, strong and moderate social support, getting information about mental illness from other people within the community, and severe perceived severity of mental illness were the variables that have a significant association with the intention to seek help from the traditional source of help.

Participants who believed mental illness was severe were 57% less likely to seek help from a traditional form of help. This justification for this might be that participants who think of mental illness as a severe form might take seek help from modern treatment than the traditional one. Besides, in our community, people think that the modern form of treatment for mental illness is the last preserved option for severe mental illness [11]. Besides, participants whose age group was from 25–34 years were 1.46 times at higher odds of having an intention to seek help from traditional forms of help than participants whose ages ranged from 18–24 years. This finding is in contradiction with the reported result of a study done in Malaysia that the younger age group is more likely to seek help than older aged individuals [9]. This might be due to those participants in Malaysia at their younger age were exposed to mass media and accesses information about mental illness easily at a younger age so that increased help-seeking in this age group.

Moreover, participants who had strong and moderate social support were more likely to seek help from a traditional form of help than respondents who had poor social support. This might be because in people with social support, different opinions might be given to bring people with mental illness to a traditional form of help. Finally, respondents’ who get information about mental illness from other people within the community were 63% less likely to seek help from the traditional form of help. This might be due to the community sharing the experience of their family members that mental illness might not improve if they took the patient to the traditional form of treatment. Another possible reason might be that they continue to hide their problems due to the fear of stigma and discrimination. For example, South African studies argued that the advocated treatment modality is talking about the problem of seeking professional help [14, 23]. One 29-year-old female focus group discussion participant supported this as ‘*it is a good solution if someone who has mental illness talks with his close friend and shares what is a disturbing issue’*. She considers social support as a solitary source of help for mental illness. This ideology might be the cause for those who have social support are less likely to seek help from health professionals in this study. The current finding contradicts the finding of a China study [15] which illustrated that people who have more information about mental illness are more likely to seek any type of help. Moreover, this study assesses help-seeking intention towards mental illness, so it is not assessing the specific disorders.

## Conclusion

The results of this study showed that more than three-fourths (80%) of the participants had the intention to seek help from health workers for their perceived mental illness. The factors significant associations with help-seeking intention were social support, age, source of information, perceived severity of mental illness and marital status. This result shows that intervention needs to enhance help-seeking intention like giving education about mental illness for the community using a media of high geographical coverage.

## Data Availability

All datasets used throughout this research process are available from the corresponding author on a reasonable request.

## Abbreviations

CAMI: Community Attitude to Mental Illness Inventory
Epi-info: Epidemiological information
GHSQ: General Help-Seeking Questionnaire
MAKS: Mental Health Knowledge Schedule
MI: Mental illness
MOH: Ministry of Health
SPSS: Statistical Package for Social Science
UOG: University of Gondar
WHO: World Health Organization
