# Correction to: Help-seeking intention and associated factors towards mental illness among residents of Mertule Mariam town, East Gojam Zone, Amhara Region, Ethiopia: a mixed-method study

**DOI:** 10.1186/s12991-020-00297-0

**Published:** 2020-08-27

**Authors:** Berhanu Yeshanew, Asmare Belete, Mogesie Necho, Shegaye Shumet, Zegeye Yohannis Maja, Dessie Abebaw Agnaw

**Affiliations:** 1 Department of Psychiatry, College of Medicine and Health Science, Dire Dewa University, Dire Dewa, Ethiopia; 2 Department of Psychiatry, College of Medicine and Health Science, Wollo University, Dessie, Ethiopia; 3 Department of Psychiatry, University of Gondar, Gondar, Ethiopia; 4 Amanuel Mental Health Hospital, Addis Abeba, Ethiopia; 5 University of Gondar, Gondar, Ethiopia

## Correction to: Ann Gen Psychiatry (2020) 19:12 10.1186/s12991-020-00261-y

Following publication of the original article [1], the author reported that the 4th, 5th and 6th authors were omitted from the author group. Shegaye Shumet, Zegeye Yohannis Maja and Dessie Abebaw have been added to the author group and are presented correctly in this correction article.

In addition, the new Authors’ contributions statement is given below:
